# A four-year survey (2011–2014) of West Nile virus infection in humans, mosquitoes and birds, including the 2012 meningoencephalitis outbreak in Tunisia

**DOI:** 10.1038/s41426-018-0028-y

**Published:** 2018-03-14

**Authors:** Abir Monastiri, Badereddine Mechri, Ana Vázquez-González, Meriadeg Ar Gouilh, Mohamed Chakroun, Chawki Loussaief, Maha Mastouri, Najet Dimassi, Lamjed Boughzala, Mahjoub Aouni, Jordi Serra-Cobo

**Affiliations:** 1 Laboratory of Contagious Diseases and Biologically Active Substances, LR99ES27, Faculty of Pharmacy, Avicenne Street, 5000 Monastir, Tunisia; 2 Laboratory of Arbovirus and Imported Viral Diseases, National Microbiology Center, Instituto de Salud Carlos III, 28220 Madrid, Spain; 3 Department of Infection and Epidemiology, Institut Pasteur, 75015 Paris, France; 4 Infectious Diseases Department, Fattouma Bourguiba University Hospital, 5000 Monastir, Tunisia; 5 Laboratory of Microbiology, Fattouma Bourguiba University Hospital, 5000 Monastir, Tunisia; 6 Unit of Genetic Research, Biodiversity and Valorization of Bioresources, UR03ES09, Higher Institute of Biotechnology, Taher Hadded Avenue, 5000 Monastir, Tunisia; 7 Ministry of Agriculture-Tunis, CRDA, 5000 Monastir, Tunisia; 8 IRBIO and Department of Animal Biology, Faculty of Biology, University of Barcelona, Av. Diagonal, 08028 Barcelona, Spain

## Abstract

A West Nile virus (WNV) outbreak occurred in Tunisia between mid-July and December 2012. To assess the epidemiological features of the WNV transmission cycle, human cerebrospinal fluid samples from patients with suspected cases (*n* = 79), *Culex pipiens* mosquitoes (*n* = 583) and serum specimens from domestic and migratory birds (*n* = 70) were collected for 4 years (2011–2014) in the Tunisian Sahel region. Viral testing was performed by polymerase chain reaction (PCR). The WNV genome was detected in 7 patients (8.8%), 4 *Culex pipiens* pools, and a domestic mallard (*Anas platyrhynchos*). All PCR-positive samples were from the Monastir region. Phylogenetic analysis revealed that two different WNV strain groups circulated, and isolates from the reservoir (bird), vector (*Culex pipiens*), and dead-end hosts (humans) were closely related. The Monastir region is a hot-spot for WNV infection, and the reiterative presence of WNV over the years has increased the risk of viral reemergence in Tunisia, which highlights the need for more enhanced and effective WNV surveillance in humans with public awareness campaigns strengthened by monitoring mosquitoes and maintaining avian surveillance for early detection of WNV circulation.

## Introduction

The West Nile virus (WNV) is an arthropod-borne virus of the *Flaviviridae* family, genus *Flavivirus*, belonging to the Japanese encephalitis serocomplex and has a complex life cycle, involving several bird species as primary hosts, mosquitoes as primary vectors and humans, and horses as incidental or dead-end hosts^1,2^. WNV is maintained in nature via native birds who serve as the local amplifying hosts, as well as infected migratory birds who spread the virus intercontinentally^3^. Most human WNV infections are subclinical or asymptomatic; however, symptomatic persons may experience a mild influenza-like illness that induces fever, headache, myalgia, malaise, vomiting, and diarrhea. Less than 1% of infected patients develop neuroinvasive diseases, including meningitis, encephalitis, or acute flaccid paralysis, with some fatalities occurring in elderly and immunocompromised people^4–6^.

In recent decades, WNV has expanded geographically and is now endemic in Southern and Eastern Europe, Africa, North America, West Asia, the Middle East and Australia^7^. WNV circulation in the Mediterranean basin has been confirmed, and large human WNV infection outbreaks continue to be reported^8,9^. In Tunisia, WNV caused three major human epidemics with fatalities and severe central nervous system diseases, such as meningitis and encephalitis, which occurred in 1997^10,11^, 2003^5,12^, and more recently in 2012^6^, with sporadic cases recorded in 2007, 2010, 2011, and 2016^9,13,14^. The WNV strains identified during 1997 and 2003 human outbreaks belonged to lineage 1a and were closely related to the Israeli-American cluster^5,11,15^. WNV is thought to be endemic/enzootic in Tunisia, as shown by several serological studies performed in humans^16–18^, wild birds^19^, and other mammals^20–23^. Furthermore, WNV has been detected in mosquitoes from Central Tunisia^24^.

The Tunisian Sahel is a coastal region of Eastern Tunisia near Malta and the Sicilian Islands. It stretches along the eastern shore of three governorates (Sousse, Monastir, and Mahdia). The Sahel region was affected by the three WNV epidemics, and its governorates are considered hotspots^22^ based on avifauna biodiversity and the regional abundance of wetlands and mosquito populations, primarily *Culex* species, which are potential WNV vectors in Tunisia^25,26^. Moreover, the Sahel region is the resting and wintering place of many migratory and native bird species, which could be a risk factor for WNV infection^25,27^. Thus, monitoring *Flavivirus* infections in this coastal region is important for public health.

In the present study, we report outcomes of the first molecular WNV characterization from clinical samples from patients who had suspected WNV infections or neurological symptoms during the 2012 outbreak in the Tunisian Sahel region. The study also provides a survey of mosquitoes and birds conducted to compare the WNV strains circulating in wildlife and humans and to obtain epidemiological information about the WNV transmission cycle in the Tunisian Sahel region.

## Results

### Patient clinical characteristics and WNV detection

Seventy-nine patients (48 males and 31 females) from 22 localities in the Tunisian Sahel region were admitted to Fattouma Bourguiba University Hospital of Monastir (Tunisia) and distributed as follows: 19 infants (24%), 18 children (23%), 36 adults (45%), and 6 elderly patients (8%) (Table 1). The mean patient age was 21.42 years (range: 1 month–76 years).

**Table 1:** Distribution of patients and confirmed West Nile virus cases by year of sampling in the Monastir region, Tunisia

	2012				2013				2014			
	A	B	C	D	A	B	C	D	A	B	C	D
Women	11	4 (36%)			12	1 (8%)			8	0		
Men	21	1 (5%)			16	1 (6%)			11	0		
Total	32	5 (16%)	15	5 (34%)	28	2 (7%)	13	2 (15%)	19	0	11	0

*A* no. of patients analyzed, *B* no. and % of WNV-positive patients, *C* no. of localities analyzed, *D* no. of localities positive for WNV and % of those analyzed

**Fig. 1:**
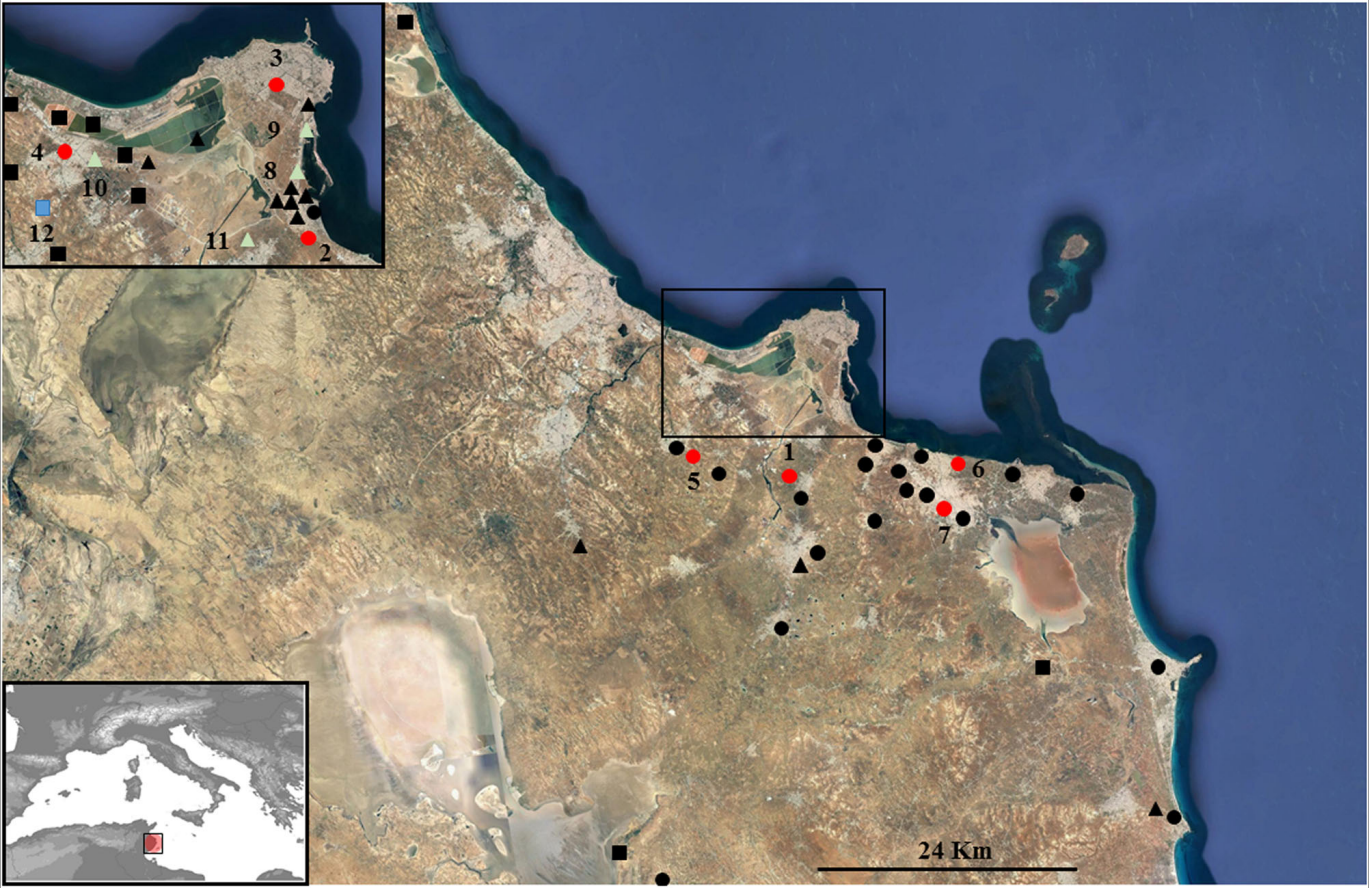
Geographic distribution of PCR-positive human, avian and pooled mosquito samples in the Monastir governorate, Sahel region, Tunisia. 1, Bembla; 2, 8, Kniss; 3, Monastir; 4, Moôtmar; 5, Ouardanine; 6, Sayada; 7, Ksar Helal; 9, Agba; 10, Moôtmar- Sahline; 11, Oued khniss; 12, Ouardanine Oued el Guelta. Red circles: positive human samples. Black circles: negative human samples. White triangles: positive mosquito pools. Black triangles: negative mosquito pools. Blue square: positive bird sample. Black squares: negative bird samples

Among the 79 cerebrospinal fluid (CSF) samples, 7 (8.8%) were positive for WNV RNA (5 females and 2 males) originating from 7 localities (Fig. 1): 4 and 1 were obtained in November and December 2012, respectively, and 2 in January 2013 (Table 2). Four patients had neuroinvasive diseases: three had meningitis and one meningoencephalitis with a past medical history of hypertension, one patient experienced febrile seizures and two patients died. All patients received supportive treatment, and the brain computed tomography scan of the meningoencephalitis patient was normal.

**Table 2:** Clinical and demographic data and virological findings of the seven West Nile virus-positive patients in the Monastir region, Tunisia

Patient	Sample	Year	Virus	Sex	Age (years)	Fever (°C)	Symptoms	Locality	Geographic coordinates
1	CSF	2012	HS_1	Ma	65.00	39.00	C, F, Nr, M	Bembla	35°40′55.10″N/10°45′58.19″E
2	CSF	2012	HS_5	F	50.00	38.60	F, Nr, Gp, ME	Khniss	35°43′5.28″N/10°48′50.79″E
3	CSF	2012	HS_7	F	31.00	38.50	C, F, Nr, V, M	Monastir	35°45′51.31″N/10°48′40.64″E
4	CSF	2012	HS_3	F	0.90	38.70	Sh, D, F, V	Moôtmar	35°45′4.29″N/10°41′27.23″E
5	CSF	2012	HS_6	F	1.00	38.00	F, S	Ouardanine	35°42′31.36″N/10°40′43.66″E
6	CSF	2013	HS_2	Ma	66.00	—	A, C	Sayada	35°39′40.80″N/10°54′15.94″E
7	CSF	2013	HS_4	F	36.00	39.00	C, F, Nr, V, M	Ksar Helal	35°38′42.94″N/10°52′24.14″E

*CSF* cerebrospinal fluid, *HS* human sample, *Ma* male, *F* female, *A* asthenia, *C* headache, *Sh* shock, *S* seizures, *D* diarrhea, *F* fever, *Gp* general physical deterioration, *Nr* nuchal rigidity, *V* vomiting, *M* meningitis, *ME* meningoencephalitis

### WNV detection in mosquitoes

A total of 583 *Culex* (*Cx.) pipiens* female mosquitoes were collected and grouped into 25 pools (Table 3). Among the 25 mosquito pools tested for WNV RNA, 4 pools sampled in 4 localities (Khniss, Agba, Moôtmar-Sahline, Oued Khniss) in the Monastir region were positive by PCR (Fig. 1). Mosquitoes from 1 and 3 of the WNV-positive pools were captured in October 2011 and November 2012, respectively.

**Table 3:** Features of *Culex pipiens* mosquitoes (*n* = 583) sampled for West Nile virus testing in the Sahel region, Tunisia

No. of females analyzed	Capture day	Locality	Geographic coordinates	nRT-PCR results
45	2011/10/03	Khniss	35°43′13.08″N/10°49′11.11″E	MS_8
7	2011/10/25	Khniss	35°43′4.06″N/10°48′50.91″E	Negative
15	2011/10/31	Khniss	35°43′21.0″N/10°49′12.84″E	Negative
Total 2011 = 67				
25	2012/09/07	Oued khniss	35°43′21.45″N/10°48′33.95″E	Negative
46	2012/09/15	Oued khniss		Negative
19	2012/11/01	Agba	35°45′19.31″N/10°49′39.04″E	MS_9
12	2012/11/08	Sahline aéroport	35°44′39.29″N/10°44′15.86″E	Negative
9	2012/11/12	Moôtmar- Sahline	35°45′10.70″N/10°42′16.47″E	MS_10
19	2012/11/17	Oued khniss	35°43′18.44″N/10°48′50.28″E	Negative
7	2012/11/18	Oued khniss		Negative
12	2012/11/21	Oued khniss		MS_11
7	2012/11/22	Oued khniss		Negative
9	2012/11/24	Oued khniss		Negative
Total 2012 = 165				
24	2013/09/02	Oued khniss	35°43′18.44″N/10°48′50.28″E	Negative
20	2013/09/15	ONAS Ouardanine	35°43′12.38″N/10°40′25.19″E	Negative
29	2013/09/27	Agba	35°45′3.98″N/10°49′38.54″E	Negative
34	2013/09/01	Oued Hamdoun	35°46′45.17″N/10°41′0.28″E	Negative
33	2013/09/03	Oued Hamdoun		Negative
45	2013/09/14	Oued Hamdoun		Negative
30	2013/09/15	Sahline aéroport	35°44′39.29″N/10°44′15.86″E	Negative
18	2013/10/01	Jemmel	35°37′30.96″N/10°46′0.70″E	Negative
Total 2013 = 233				
25	2014/09/01	Mahdia	35°25′5.35″N/11° 0′49.28″E	Negative
20	2014/09/15	Mahdia		Negative
34	2014/09/30	Mahdia		Negative
39	2014/11/01	Frina	35°43′36.54″N/10°48′59.62″E	Negative
Total 2014 = 118				

*nRT-PCR* nested reverse transcription-polymerase chain reaction, *MS* mosquito sample

### WNV detection in migratory and domestic birds

Among the 70 bird serum samples, only one bird tested positive for WNV RNA: a domestic mallard (*Anas platyrhynchos*) sampled in November 2013 from a livestock farm in Ouardanine-Oued El Guelta in the Monastir region (Fig. 1; Table 4).

**Table 4:** West Nile virus surveillance findings on domestic (*n* = 23) and migratory birds (*n* = 47) sampled in the Sahel region, Tunisia

Species	D/W	A/B	Capture day	Locality	Geographic coordinates	nRT-PCR results
Cormorant	W	0/6	2012/10-11	Sahline-Sabkha- Airport	35°45′27.67″N/10°42′59.54″E	Negative
Egret	W	0/3	2012/10-11	Sahline-Sabkha- Airport		Negative
Pintail	W	0/3	2012/11/22	Sahline-Sabkha- Airport		Negative
Snipe	W	0/3	2012/11/30	Sahline-Sabkha- Airport		Negative
Spoonbill	W	0/3	2012/11/30	Sahline-Sabkha- Airport		Negative
European herring gull	W	0/5	2013/09/13	Sahline-Sabkha- Airport		Negative
European herring gull	W	0/2	2012/12/12	Ouardanine	35°42′34.74″N/10°40′32.82″E	Negative
European starling	W	0/3	2012/12/12	Mesjed Aïssa	35°43′36.38″N/10°43′5.67″E	Negative
European starling	W	0/3	2013/01/16	Mesjed Aïssa		Negative
European herring gull	W	0/5	2013/10/28	Saline-Sahline	35°45′47.34″N/10°42′25.72″E	Negative
White duck	D	0/4	2013/11/01	Mahdia	35°30′32.19″N/10°57′49.17″E	Negative
Barbary duck	D	0/3	2013/11/12	Irrigation area of Sahline	35°44′6.95″N/10°42′12.08″E	Negative
Chickens	D	0/3	2013/11/12	Irrigation area of Sahline		Negative
Gray goose	D	0/2	2013/11/12	Sahline-Sabkha- Airport	35°44′38.72″N/10°44′16.01″E	Negative
Hen	D	0/3	2013/11/12	Sahline-Sabkha- Airport		Negative
European starling	W	0/4	2013/11/22	Oued Hamdoun	35°46′27.35″N/10°41′18.63″E	Negative
Wild duck	D	1/2	2013/11/25	Ouardanine Oued el Guelta	35°43′35.18″N/10°40′25.48″E	BS_12
Gray goose	D	0/2	2013/11/25	Ouardanine Oued el Guelta		Negative
European herring gull	W	0/1	2013/11/28	Mahdia	35°24′50.80″N/10°34′3.45″E	Negative
European herring gull	W	0/1	2013/11/29	Mahdia		Negative
Seagull	W	0/4	2013/11/29	Mahdia		Negative
Egret	W	0/1	2013/11/30	Mahdia		Negative
Wild duck	D	0/2	2013/12/07	Hergla Foukaïa	36° 1′11.21″N/10°30′5.51″E	Negative
Gray goose	D	0/2	2013/12/07	Hergla Foukaïa		Negative

*A* number of positive birds, *B* number of birds analyzed, *D* domestic, *W* wild, *nRT-PCR* nested reverse transcription-polymerase chain reaction, *BS* bird sample

### Phylogenetic analysis

**Fig. 2:**
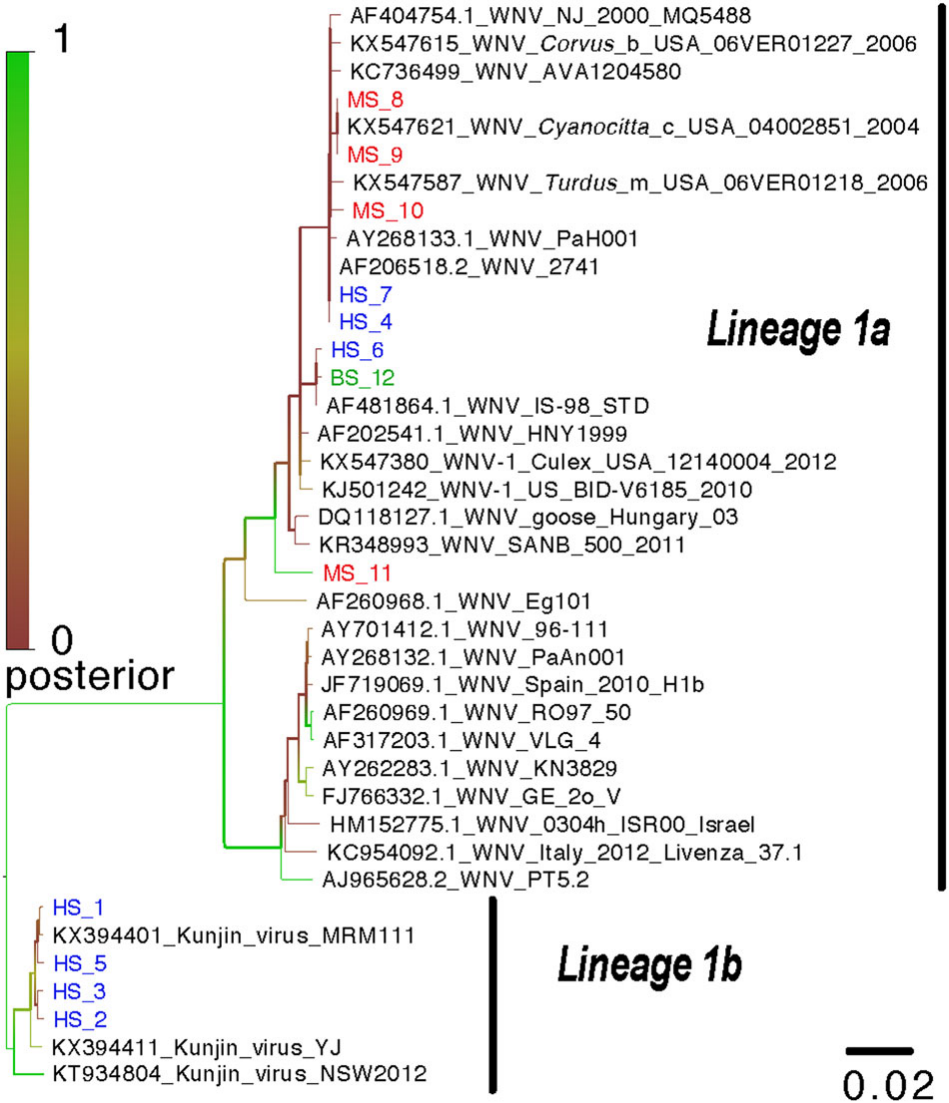
Phylogenetic analysis of identified West Nile virus sequences from humans, pooled mosquitoes and birds in the Monastir governorate, Sahel region, Tunisia. Phylogenetic analysis was performed using Bayesian analysis based on the TN93 evolution model, a gamma distribution and invariable sites. The tree includes 12 strains isolated in this study and 27 homologous nucleotide sequences from the West Nile virus *NS5* gene obtained from the GenBank library. HS human sample, MS mosquito sample, BS bird sample

A phylogenetic tree was constructed from a 283-bp section of the *NS5* gene region amplified by the WNV primers (WNV2+/WNV2−). The tree including the 12 WNV isolates from this study and their relationship with other WNV strains is presented in Fig. 2. As shown in the figure, only strains from lineage 1 were detected. The WNV-positive PCR amplicons obtained from three patients, the bird and the 4 mosquito pools formed a monophyletic cluster and were closely related to a Tunisian WNV strain isolated in 1997 (PAH001) as well as the Israeli strain, IS-98. The 4 remaining human sequences formed a second distinct cluster and belonged to the Kunjin strains group (Lineage 1b).

Sequences obtained were submitted in GenBank under the accession number, MF371349-60.

## Discussion

During the summer-autumn of 2012, a WNV infection outbreak occurred in Tunisia after an apparent silent period following the 2003 epidemic, although epidemiological and clinical surveys have demonstrated WNV circulation in humans^5,10–12^, as well as in mosquitoes^24^, and seropositivity has been reported in equids^20–22^, dromedaries^23^, and wild birds^19^ in the years since the first WNV epidemic in 1997. However, to date, no information on the epidemiological characteristics of the WNV transmission cycle in the Tunisian Sahel region has been available. This coastal region of Tunisia is interesting to study from an epidemiological viewpoint as (i) the first WNV human cases during 1997, 2003, and 2012 outbreaks have been reported in the Tunisian Sahel region; (ii) it is located on the northern coast of Africa across the Mediterranean Sea from Italy and could therefore serve as a gateway for birds migrating between Africa and Europe; and (iii) wetlands identified in the Tunisian Sahel region are overwintering and resting areas for several bird species.

On the basis of our WNV RNA detection results from human samples, the WNV prevalence (8.8%) was low and is consistent with a previous study that reported WNV genome detection in clinical specimens by nested PCR (8.8%) during the 2003 meningitis/meningoencephalitis epidemic in the Monastir region^5^.

Phylogenetic analysis revealed that our WNV isolates belonged to two distinct clusters: human, avian and mosquito isolates closely related to the virulent WNV strains isolated in Tunisia (1997) and in Israel (1998), suggesting the presence of an active and local WNV transmission cycle in the Monastir area. The second cluster comprised four isolates from human samples possibly related to the Kunjin virus. These findings revealed the emergence of new pathogenic WNV strains different from those circulating in the Monastir area during the 2003 outbreak^5^. Two of the four patients whose CSF samples tested positive for Kunjin virus RNA presented with neuromeningeal diseases: the elderly and adult patient had meningitis and meningoencephalitis with a past medical history of hypertension, respectively. Kunjin virus detection in patients with neurological manifestations raises the question of whether risk factors have a role in the disease severity since the Kunjin virus is considered less virulent than other WNV types^28^. The contributing factors of age and chronic diseases may relate to a decreased blood–brain barrier integrity, which facilitates access of such a neurotropic virus to the central nervous system, and therefore, predisposes infected patients to neurological complications^29^.

Per the National Observatory of New and Emerging Diseases (Tunisia), the 2012 WNV epidemic occurred earlier in Tunisia from mid-July and persisted beyond the usual occurrence period to December with a wider geographical spread in addition to an increasing number of reported neuroinvasive disease cases and deaths compared to the two previous epidemics of 1997 and 2003, which likely indicates a stronger WNV dynamic in Tunisia in 2012^13,30^. This epidemic coincided with WNV emergence in several other European countries^31–34^ and the United States^35^, who also reported earlier occurrences of an increased number of human WNV infection cases. In our study, among the seven WNV-positive cases, four were obtained in November 2012, one in December 2012 and two in January 2013, while WNV was undetected in patients during the transmission seasons of 2013 and 2014. Fewer WNV human cases and small-scale epidemics were reported in 2014 in Southern and Central Europe, as well as in the Middle East, compared to the exceptional transmission seasons of 2012 and 2013, which could be linked to the summer weather conditions in 2014 (temperature and precipitation), which were unfavorable for viral maintenance and amplification^36,37^.

The seven WNV-positive cases originated from seven localities (Monastir, Bembla, Khniss, Ouardanine, Moôtmar, Ksar Helal, Sayada) in the Monastir governorate. Notably, the Monastir area is a former hotbed of WNV infection and was the first governorate affected by the 2012 epidemic^13^. The presence of a local and active WNV transmission cycle in this region suggests that Monastir is a high-risk area for WNV infection as demonstrated by previous epidemiological studies^22,38^.

In the current study, WNV-positive cases included three patients with non-specific non-neurological clinical manifestations and four patients with laboratory-confirmed central nervous system infections. However, even without neurological manifestations, WNV infection can cause significant public health problems, as patients such as children and workers who sought medical attention could not attend school or work because of the illness^39^. Only hospitalized patients were included in this study. Thus, WNV can cause mild self-limited febrile manifestations that may not require hospitalization; therefore, the overall disease incidence may be underestimated.

The roles of migratory birds as introductory hosts, as well as indigenous wild and domestic birds as potential WNV-circulating reservoirs, have previously been discussed^40,41^, and several attempts to isolate the virus from birds have been documented^42–44^. A domestic mallard (*Anas platyrhynchos*) sampled in November 2013 from a livestock farm in Ouardanine-Oued El Guelta in the Monastir region, tested positive for WNV RNA. This sampling site was chosen based primarily on a serological survey conducted at the same farm that showed evidence of WNV antibodies in domestic ducks and highlighted WNV circulation in this area; however, no WNV genomic RNA was detected^45^. Asian studies showed the receptivity and susceptibility of ducks to WNV infection as well as the birds’ involvement in WNV transmission^46,47^. Our results suggest that this WNV-positive domestic duck was viremic at the time of sampling, and could therefore act as a WNV reservoir; thus, it may have been involved in the local WNV transmission cycle. However, per Hofmeister et al.^48^, it is unlikely that ducks are amplifying hosts or that they play significant roles in WNV transmission. The homology between the sequences obtained from this bird and the mosquitoes suggests that the virus is overwintered locally or reintroduced seasonally, and the environment favors viral amplification. In a similar study conducted in Italy, WNV persistence was also confirmed through viral circulation in birds and mosquitoes^43^. Furthermore, the studied area is situated near wadi El Guelta with approximately 5 km of wetlands, namely Sahline-Sabkha-Airport and Saline-Sahline, with high concentrations of migratory birds, some of which were sampled and included in this study. Although WNV was undetected in the 47 migratory bird samples, the likely contribution of viremic migratory birds in repeatedly introducing WNV in Tunisia, followed by local viral transmission via amplifying avian hosts, has been clearly assessed in many Tunisian studies^10,12,19^.

*Cx. pipiens* mosquitoes are considered the most important vectors in transmitting WNV^43,49,50^. Entomological surveys conducted after WNV outbreaks in Tunisia showed the abundance and role of *Cx. pipiens* as potential WNV vectors in Tunisia^11,24,26,51,52^. WNV-positive *Cx. pipiens* pools sampled in 4 localities (Khniss, Agba, Moôtmar-Sahline, and Oued-Khniss) in the Monastir governorate indicated viral circulation during the 2011 and 2012 seasons. Despite no human cases having been reported in the Tunisian Sahel region in 2011^53^, our findings strongly suggest a silent WNV circulation during the 2011 transmission season and that the onset of the 2012 WNV outbreak was favored by ecological factors and environmental conditions. The epidemic occurred during a very hot summer season with a high rainfall, which provided optimal conditions for mosquito proliferation. Interestingly, such exceptionally warm climate conditions were observed during the two previous epidemics in Tunisia^25^, as well as in 2012 during the WNV outbreaks in Europe^31,33,34^ and the United States^54^. Although WNV was undetected in *Cx*. *pipiens* mosquitoes collected in 2013 and 2014, a study conducted in central Tunisia reported viral detection in *Cx. pipiens* during the 2014 transmission season suggesting WNV persistence in the country^24^. More studies are needed regarding vector competence and overwintering cycles in mosquitoes, local birds and potential non-avian reservoirs^23^ as an endemization mechanism in Tunisia.

Isolate clustering from human and mosquito WNV-positive samples in this study confirms the potential role of *Cx. pipiens* as a bridge species between birds and mammals, and its involvement in the WNV transmission cycle in Tunisia has been described in several Tunisian entomological studies^24,26,52^. WNV detection in both human and *Cx. pipiens* specimens sampled in the Monastir region during the 2012 outbreak suggests that after WNV amplification via an enzootic transmission cycle, human exposure to infected mosquitoes and the viral shift to humans may have occurred under ecological and environmental conditions including the close proximity of human habitats to wetlands that host migratory bird settlements and are suitable areas for mosquitoes^55^. All patient and *Cx. pipiens* mosquito specimens in this study sampled during the 2013 and 2014 transmission seasons were WNV-negative. However, WNV detection in birds in 2013 (this study) and in mosquitoes in 2014^24^ suggest that the risk of WNV reemergence in Tunisia should be considered high.

As no vaccine or specific treatment exists for WNV infections, only preventive measures, such as wearing long-sleeve shirts and long pants, applying skin repellents and avoiding being outdoors at dusk could reduce the risk of WNV infection by half^56^. Although vector control measures including adulticidal and larvicidal treatments were implemented early in Tunisia in spring 2012, it appears that the population’s low background immunity to WNV and the emergence of new pathogenic strains may partly explain the reoccurrence of WNV human cases and the spread of the virus to new areas^13,16^. Thus, human surveillance should be enhanced by updating risk area maps and public awareness campaigns among the population and health care professionals throughout the country. WNV persistence may lead to future outbreaks in Tunisia and along bird migratory routes between Africa and Europe, highlighting the need for entomological studies on persistence mechanisms and identification of mosquito species acting as competent bridge vectors in WNV transmission^24^. Setting up avian surveillance based on serological surveys of domestic birds cohabiting with humans and/or sentinel chickens to assess WNV enzootic transmission might provide useful epidemiological information such as low-noise WNV circulation, which can therefore be used as a warning system to detect viral circulation early and implement prevention and control measures^57,58^.

## Materials and methods

### Ethics statement

The study protocol was approved by the Ethics and Research Committee of the Fattouma Bourguiba University Hospital (Monastir, Tunisia, Committee’s advice on 20 September 2013), and written informed consent was obtained from the 79 patients.

### Patients and clinical sample collections

Seventy-nine CSF samples were obtained from patients admitted to different departments of the Fattouma Bourguiba University Hospital (Monastir, Tunisia) from November 2012 to December 2014. Per routine clinical care, lumbar punctures were performed by the examining physician on patients showing signs of suspected acute meningitis upon admission at the emergency department or within 24 h of hospitalization. CSF specimens were processed for microbiological testing by routine hospital procedures and were bacterial pathogen-free. CSF cytobiochemical examinations provided pleocytosis as well as protein and glucose concentrations.

Based on the examining physician’s clinical diagnosis and medical records, patients with febrile symptoms and/or exhibiting suspicious signs of neuromeningeal infection, such as aseptic meningitis, encephalitis, and meningoencephalitis, were included in the study. Complete demographic characteristics and clinical findings were obtained for all patients.

### Study sites and mosquito collections

Mosquito sampling was performed at different sites in the Sahel region’s 3 coastal governorates, Monastir, Sousse, and Mahdia, mostly from September to November for 4 years (2011–2014). Two battery-powered CDC light-traps were placed at 14 sampling sites and operated approximately from sunset to sunrise. Epidemiological characteristics were considered for the sampling site selection including occurrence of WNV human cases, proximity to wetlands and migratory bird settlement flight. Mosquito traps were placed in urban (houses and gardens) and rural (riding stables and livestock farms) environments.

Field-collected specimens were transported to the laboratory, and female *Cx*. *pipiens* were morphologically identified using identification keys^59^. Female *Cx. pipiens* were then pooled by date and collection site by handling mosquitoes individually with sterile stainless steel tweezers with a maximum of 50 individuals per pool and stored in cryotubes at −70 °C until being assayed for viral detection.

Prior to nucleic acid extraction, mosquito pools were homogenized following a procedure described previously^60^.

### Bird collections

Seventy serum samples from 23 domestic and 47 migratory birds were obtained from 2012 to 2014 during the autumn season and active mosquito circulation periods. Migratory bird blood sampling was conducted at several bird wintering and resting sites. Domestic bird blood was collected from livestock farms located on wild bird flyways and/or close to wetlands in the Tunisian Sahel region. For each bird, 5 ml of blood was obtained and centrifuged at 12 000 rpm for 10 min. Serum samples were stored at −20 °C until tested.

### Detection of WNV RNA

Total RNA was extracted from 200-µl samples using the Trizol^®^ LS Reagent (Sigma-Aldrich, Madrid, Spain) method for CSF samples and mosquito pool supernatants. Sera from domestic and migratory birds were processed using Trizol^®^ BD Reagent (Sigma-Aldrich, Madrid, Spain). RNA was then purified with chloroform and precipitated with isopropanol (Sigma-Aldrich, Madrid, Spain).

After washing with 70% ethanol, the pellet was dried and eluted in 30 µl of RNase-free water (Qiagen, Barcelona, Spain). A negative control consisting of RNase-free water was included in this step.

Generic RT-PCR was performed using degenerated primers, Flavi1+ and Flavi1−, whereas generic nested PCR was performed using degenerated primers, Flavi2+ and Flavi2−, as previously described^61^. These two primer sets were designed to amplify a conserved region of the flavivirus genome located in the *NS5* gene encoding for polymerase. A second generic nested PCR was performed using degenerated primers designed to specifically target the *NS5* gene of the WNV genome (WNV2+: 5′_8485_AARCCYCTNCTYAAYTCWGAYAC3′_8507_ /WNV2−: 5′_8813_ TCRTTSARNACNWRYTTIRCWCC3′_8791_).

The RT-PCR step was performed using the One Step RT-PCR kit (Qiagen, Barcelona, Spain) per the manufacturer’s instructions using 10 µl of RNA and 100 pmol of each primer (Flavi1+/Flavi1−) in a 50-µl total reaction volume. Samples underwent an initial cycle at 50 °C for 30 min and 95 °C for 15 min, followed by 40 PCR cycles at 94 °C for 30 s, 40 °C for 4 min, 72 °C for 1 min, and a final elongation step at 72 °C for 10 min.

The nested-PCR reaction was performed using the Taq PCR Core kit (Qiagen, Barcelona, Spain) per the manufacturer’s instructions. One microliter of the first amplification product was added to 100 pmol of the second primer set (Flavi2+/Flavi2−) in a final volume of 50 µl. The reaction mixture was then subjected to the following amplification conditions: an initial denaturation step at 94 °C for 2 min followed by 40 PCR cycles at 94 °C for 30 s, 50 °C for 1 min, 72 °C for 30 s, and a final elongation step at 72 °C for 10 min. The expected PCR product size was 143 bp.

For the second nested WNV-specific PCR reaction, 1 µl of the RT-PCR product was added to 50 µl of the total reaction mixture containing 100 pmol of each primer (WNV2+/WNV2−) with the following cycling conditions: 94 °C for 3 min and 40 PCR cycles at 94 °C for 1 min, 45 °C for 3 min, 72 °C for 1 min, and a final elongation step at 72 °C for 10 min. The expected amplicon size was 328 bp.

After the amplifications, 10 µl of nested-PCR product was loaded into a 2% GelRed (Biotium) stained agarose electrophoresis gel in TBE buffer and visualized under ultraviolet light. Amplicon sizes were determined by comparing them with a 100-bp DNA ladder (Qiagen, Barcelona, Spain).

### Sequencing and phylogenetic analysis

PCR products were purified using the QIAquick PCR purification kit (Qiagen, Barcelona, Spain) and bi-directionally sequenced (Macrogen Inc., Amsterdam, the Netherlands). The phylogenetic analysis (Bayesian analysis) was conducted using the beast package^62^, with the TN93 evolution model, a gamma distribution and invariable sites. The clock parameters were set to uncorrelated lognormal, using a coalescent constant size model. The chain length was set at 10 million iterations to produce an ESS (effective sampling size) superior to 200. The maximum credibility tree with branch length in number of substitutions was defined from ten thousand trees after discarding 10% and was edited using FigTree (http://tree.bio.ed.ac.uk/software/figtree/).

## References

[1] Thiel, H. J, Collett, M. S. & Gould, E. A. et al. in *Virus Taxonomy. VIIIth Report of the International Committee on Taxonomy of Viruses (ICTV)* (eds Fauquet, C. M., Mayo, M. A., Maniloff, J., Desselberger, U. & Ball, L. A.) 979–996 (Elsevier/Academic Press, London, 2005).

[2] Blitvich BJ (2008). Transmission dynamics and changing epidemiology of West Nile virus. Anim. Health Res. Rev..

[3] Rappole JH, Hubálek Z (2003). Migratory birds and West Nile virus. J. Appl. Microbiol..

[4] Kramer LD, Li J, Shi PY (2007). West Nile virus. Lancet Neurol..

[5] Riabi S, Gaaloul I, Mastouri M (2014). An outbreak of West Nile Virus infection in the region of Monastir, Tunisia, 2003. Pathog. Glob. Health.

[6] Bouatef S, Hogga N, Ben Dhifallah I (2012). Monitoring and current situation of meningitis and meningoencephalitis to West Nile virus in Tunisia. Tun. Rev. Infect..

[7] World Health Organization. *West Nile Virus. Media Centre. Fact sheet No. 354*. (WHO, Geneva, 2011). http://www.who.int/mediacentre/factsheets/fs354/en/. Accessed July 2011.

[8] Di Sabatino, D., Bruno R. & Sauro, F. et al. Epidemiology of West Nile disease in Europe and in the Mediterranean Basin from 2009 to 2013. *Biomed. Res. Int*. **2014**, 907852 (2014). 10.1155/2014/907852 PMC4180897 25302311

[9] European Centre for Disease Prevention and Control. *Epidemiological Update: West Nile Virus Transmission Season in Europe* (ECDC, Solna, 2016). https://ecdc.europa.eu/en/news-events/epidemiological-update-west-nile-virus-transmission-season-europe-2016. Accessed 15 December 2016.

[10] Triki H, Murri S, Le Guenno B (2001). West Nile viral meningoencephalitis in Tunisia. Med Trop..

[11] Feki I, Marrakchi C, Ben Hmida M (2005). Epidemic West Nile virus encephalitis in Tunisia. Neuroepidemiology.

[12] Hachfi W, Bougmiza I, Bellazreg F (2010). Second epidemic of West Nile virus meningoencephalitis in Tunisia. Med Mal. Infect..

[13] Observatoire National des Maladies Nouvelles et Emergentes. Bilan de la surveillance des Infections à Virus West Nile en Tunisie, Année 2012 (ONMNE, Tunis, 2013). http://www.onmne.tn/fr/images/Bulletin2WN.pdf. Accessed April 2013.

[14] Benjelloun A, El Harrak M, Belkadi B (2016). West Nile disease epidemiology in North-West Africa: bibliographical review. Transbound. Emerg. Dis..

[15] Charrel RN, Brault AC, Gallian P (2003). Evolutionary relationship between Old World West Nile virus strains. Evidence for viral gene flow between Africa, the Middle East, and Europe. Virology.

[16] Riabi S, Gallian P, Gaaloul I (2010). Prevalence of IgG antibodies against West Nile virus in blood donors during the 2003 outbreak in Tunisia. Trans. R. Soc. Trop. Med. Hyg..

[17] Bahri O, Dhifallah I, Ben Alaya-Bouafif N (2011). Sero-epidemiological study of West Nile virus circulation in human in Tunisia. Bull. Soc. Pathol. Exot..

[18] Kooli I, Loussaif C, Ben Brahim H (2017). West Nile virus (WNV) presenting as acute cerebellar ataxia in an immunocompetent patient. Rev. Neurol..

[19] Hammouda A, Lecollinet S, Hamza F (2015). Exposure of resident sparrows to West Nile virus evidenced in South Tunisia. Epidemiol. Infect..

[20] Ben Hassine T, Hammami S, Elghoul H (2011). Detection of circulation of West Nile virus in equine in the north-west of Tunisia. Bull. Soc. Pathol. Exot..

[21] Ben Hassine T, De Massis F, Calistri P (2014). First detection of co-circulation of West Nile and Usutu viruses in equids in the south-west of Tunisia. Transbound. Emerg. Dis..

[22] Bargaoui R, Lecollinet S, Lancelot R (2015). Mapping the serological prevalence rate of West Nile fever in equids, Tunisia. Transbound. Emerg. Dis..

[23] Hassine TB, Amdouni J, Monaco F (2017). Emerging vector-borne diseases in dromedaries in Tunisia: West Nile, bluetongue, epizootic haemorrhagic disease and Rift Valley fever. Onderstepoort J. Vet. Res.

[24] Wasfi F, Dachraoui K, Cherni S (2016). West Nile virus in Tunisia, 2014: first isolation from mosquitoes. Acta Trop..

[25] El Ghoul H. Renforcement de la surveillance et des systèmes d’alerte pour la fièvre catarrhale ovine, la fièvre du Nil Occidental et la rage au Maroc, en Algérie et en Tunisie – Fièvre du Nil Occidental: historique et situation épidémiologique en Tunisie. Projet GCP/RAB/002/FRA, FAO. 24 p. (2009).

[26] Krida G, Rhim A, Daaboub J (2015). New evidence for the potential role of Culex pipiens mosquitoes in the transmission cycle of West Nile virus in Tunisia. Med Vet. Entomol..

[27] Hamdi N, Chard-Cheikhrouha F (2011). Estimation du nombre total des oiseaux aquatiques hivernant en Tunisie: période 2001/2002 à 2006/2007. Rev. Ecol..

[28] Garcia-Sastre, A. & Endy, T. P. in *Encyclopedia of Microbiology* (ed. Moselio, S.) 3rd edn, 313–320 (Elsevier/Academic Press, Oxford, 2009).

[29] Hall RA, Broom AK, Smith DW (2002). The ecology and epidemiology of Kunjin virus. Curr. Top. Microbiol Immunol..

[30] EpiSouth. Weekly Epi Bulletin—N°239 10th October—17th October 2012. http://www.episouthnetwork.org/sites/default/files/bulletin_file/eweb_239_18_10_12.pdf.

[31] Barzon L, Pacenti M, Franchin E (2013). Large human outbreak of West Nile virus infection in north-eastern Italy in 2012. Viruses.

[32] Popović N, Milošević B, Urošević A (2013). Outbreak of West Nile virus infection among humans in Serbia, August to October 2012. Eur. Surveill..

[33] Pem-Novosel I, Vilibic-Cavlek T, Gjenero-Margan I (2014). First outbreak of West Nile virus neuroinvasive disease in humans, Croatia, 2012. Vector Borne Zoonotic Dis..

[34] Pervanidou D, Detsis M, Danis K (2014). West Nile virus outbreak in humans, Greece, 2012: third consecutive year of local transmission. Eur. Surveill..

[35] Centers for Disease Control and Prevention. *West Nile Virus (WNV) Human Infections Reported to ArboNET, by State, United States, 2012* (CDC, Atlanta, 2012). http://www.cdc.gov/westnilestatsMaps/finalMapsData/index.html. Accessed 20 February 2013.

[36] European Centre for Disease Prevention and Control. Cumulative number of West Nile fever cases by affected area in 2014 (ECDC, Solna, 2014). http://ecdc.europa.eu/en/healthtopics/west_nile_fever/West-Nile-fever-maps/Pages/2014-table.aspx. Accessed 30 November 2014.

[37] Bahuon C, Lecollinet S (2015). Des saisons de transmission du virus West Nile contrastées enEurope. Bull. Epid. St. Anim. Alim..

[38] Ben Hassine T, Conte A, Calistri P (2017). Identification of suitable areas for West Nile virus circulation in Tunisia. Transbound. Emerg. Dis..

[39] Hayes EB, Sejvar JJ, Zaki SR (2005). Virology, pathology, and clinical manifestations of West Nile virus disease. Emerg. Infect. Dis..

[40] Rappole JH, Derrickson SR, Hubálek Z (2000). Migratory birds and spread of West Nile virus in the Western Hemisphere. Emerg. Infect. Dis..

[41] Jourdain E, Zeller HG, Sabatier P (2008). Prevalence of West Nile virus neutralizing antibodies in wild birds from the Camargue area, southern France. J. Wildl. Dis..

[42] Malkinson M, Banet C, Weisman Y (2002). Introduction of West Nile virus in the Middle East by migrating white storks. Emerg. Infect. Dis..

[43] Calzolari M, Gaibani P, Bellini R (2012). Mosquito, bird and human surveillance of West Nile virus and Usutu viruses in Emilia-Romagna Region (Italy) in 2010. PLoS ONE.

[44] Maquart M, Boyer S, Rakotoharinome VM (2016). High prevalence of West Nile virus in domestic birds and detection in 2 new mosquito species in Madagascar. PLoS One.

[45] Bargaoui R. Epidémiologie de la fièvre West Nile en Tunisie. Thèse Pour obtenir le grade de Docteur de l’Université de Montpellier II. Virologie. Université de Montpellier II, Sciences et Techniques du Languedoc (2012).

[46] Shirafuji H, Kanehira K, Kubo M (2016). Experimental West Nile virus infection in aigamo ducks, a cross between wild ducks (*Anas platyrhynchos*) and domestic ducks (*Anas platyrhynchos* var. domesticus). Avian Dis..

[47] Kalaiyarasu S, Mishra N, Khetan RK (2016). Serological evidence of widespread West Nile virus and Japanese encephalitis virus infection in native domestic ducks (Anas platyrhynchos var domesticus) in Kuttanad region, Kerala, India. Comp. Immunol. Microbiol. Infect. Dis..

[48] Hofmeister E, Porter RE, Franson JC (2015). Experimental susceptibility of wood ducks (*Aix sponsa*) for West Nile virus. J. Wildl. Dis..

[49] Bagheri M, Terenius O, Oshaghi MA (2015). West Nile virus in mosquitoes of Iranian wetlands. Vector Borne Zoonotic Dis..

[50] López RH, Soto SU, Gallego-Gómez JC (2015). Evolutionary relationships of West Nile virus detected in mosquitoes from a migratory bird zone of Colombian Caribbean. Virol. J..

[51] Bouattour, A., Rhaiem, A. & Ghrammam, M. et al. Rapport d’enquête entomologique suite à l’apparition des cas de West Nile. Rapport technique, Institut Pasteur Tunis. Laboratoire d'entomologie médicale. 5 p. (1998).

[52] Amraoui F, Krida G, Bouattour A (2012). *Culex pipiens*, an experimental efficient vector of West Nile and Rift Valley fever viruses in the Maghreb region. PLoS ONE.

[53] European Centre for Disease Prevention and Control. Cumulative number of West Nile fever cases by affected area in 2011 (ECDC, Solna, 2011). http://ecdc.europa.eu/en/healthtopics/west_nile_fever/West-Nile-fever-maps/Pages/2011table.aspx. Accessed 30 November 2011.

[54] Murray KO, Ruktanonchai D, Hesalroad D (2003). Analysis of biotic and abiotic factors influencing the occurrence of West Nile virus infection in Tunisia. West Nile virus, Texas, USA, 2012.

[55] Ben Hassine T, Calistri P, Ippoliti C (2014). Analysis of biotic and abiotic factors influencing the occurrence of West Nile virus infection in Tunisia. Arch. Inst. Pasteur Tunis..

[56] Loeb M, Elliott SJ, Gibson B (2005). Protective behavior and West Nile virus risk. Emerg. Infect. Dis..

[57] Rizzoli A, Rosà R, Rosso F (2007). West Nile virus circulation detected in northern Italy in sentinel chickens. Vector Borne Zoonotic Dis..

[58] Chaintoutis SC, Dovas CI, Danis K (2015). Surveillance and early warning of West Nile virus lineage 2 using backyard chickens and correlation to human neuroinvasive cases. Zoonoses Public Health.

[59] Brunhes J, Rhaim A, Geoffroy B (2000). Les Culicidae d’Afrique Méditerranéenne.

[60] González-Reiche AS, Monzón-Pineda Mde L, Johnson BW (2010). Detection of West Nile viral RNA from field-collected mosquitoes in tropical regions by conventional and real-time RT-PCR. Methods Mol. Biol..

[61] Sánchez-Seco MP, Rosario D, Domingo C (2005). Generic RT-nested-PCR for detection of flaviviruses using degenerated primers and internal control followed by sequencing for specific identification. J. Virol. Methods.

[62] Drummond AJ, Rambaut A (2007). BEAST: Bayesian evolutionary analysis by sampling trees. BMC Evol. Biol..

